# Single nucleotide polymorphism rs854560 in paraoxonase-1 regulates the cytodifferentiation of human periodontal ligament cells

**DOI:** 10.3389/fdmed.2024.1449482

**Published:** 2024-09-20

**Authors:** Risa Masumoto, Chiharu Fujihara, Masahiro Matsumoto, Jirouta Kitagaki, Shinya Murakami

**Affiliations:** Department of Periodontology and Regenerative Dentistry, Osaka University Graduate School of Dentistry, Suita, Osaka, Japan

**Keywords:** exome sequencing, single nucleotide polymorphism, aggressive periodontitis, paraoxonase-1, periodontal ligament cells

## Abstract

Aggressive periodontitis (AgP), classified as Stages III or IV and grade C periodontitis, is characterized by the rapid destruction of periodontal tissue. Genetic factors contribute to the pathogenesis of this disease, and familial aggregation of periodontitis is often observed. However, the mechanisms underlying the onset or progression of AgP have not been elucidated. Previously, we performed exome sequencing and identified AgP risk factors in Japanese AgP-patients. However, the small sample size limited our scope for detecting some of the true AgP genetic risk factors. To overcome this limitation, we searched for AgP-related genes more comprehensively from the whole exome sequencing data of the Japanese AgP-patients by extending the filtering criteria range. We identified seven AgP-associated suggestive genes, including the single nucleotide polymorphism (SNP) rs854560 in paraoxonase-1 (*PON-1*), which is correlated with AgP. However, the mechanism(s) underlying the induction of AgP pathogenesis by the SNP rs854560 *PON-1* has not been elucidated. Thus, we further analyzed the functions of the SNP rs854560 *PON-1* in human periodontal ligament (HPDL) cells through transfection of the wild-type *PON-1* (WT) or SNP rs854560 *PON-1* (mut) into HPDL cells. Real-time PCR indicated that mut had higher mRNA expression of osteogenic related-genes and showed a higher tendency of ALP activity and proliferation. The result suggested that WT *PON-1* contributes to periodontal tissue homeostasis through appropriate proliferation and cytodifferentiation of HPDL cells, while SNP rs854560 *PON-1* may mediate excessive calcification of periodontal tissue due to hyper proliferation of HPDL cells, thereby increasing the risk of AgP.

## 1 Introduction

The periodontal ligament (PDL) is located between the tooth and the alveolar bone. It supports the teeth during mastication and provides oxygen and nutrition to the surrounding tissues (1). The PDL is composed of heterogeneous cells, called PDL cells. They contain multipotent mesenchymal stem cells that can differentiate into hard tissue-forming cells, such as cementoblasts and osteoblasts. Moreover, PDL cells play key roles in the homeostasis and healing of the periodontal tissue (2). Thus, to elucidate the pathogenesis of periodontitis, it is important to understand the mechanisms of the homeostasis and cytodifferentiation of PDL cells.

Aggressive periodontitis (AgP), defined as Stage III or IV and Grade C periodontitis in a new classification, is defined as “periodontitis characterized by the rapid destruction of periodontal tissue in those who are clinically healthy, except for periodontitis, with familial aggregation” (3). The age of onset is typically between 10 and 39 years, with little bacterial plaque in the oral cavity. The presence of familial aggregation suggests the involvement of genetic factors in the onset of periodontitis (4). Since AgP phenotypically manifests early in life, it is likely to have a higher number of risk alleles or to be caused by variants with significant effects, compared to chronic periodontitis (CP). Although single nucleotide polymorphisms (SNPs) of inflammatory cytokines have been reported as AgP-related factors, no definitive association of these SNPs with AgP has been found (5–7).

Genetic factors strongly affect susceptibility to prevalent diseases such as periodontitis and disease-related quantitative traits (8). Identifying disease-associated genes has been challenging, partially because the causal genes of this disease only make a small contribution to the overall heritability (9). Therefore, extensive information on AgP-associated genes is necessary to understand its pathogenesis. To this end, genome-wide association studies (GWAS) have been widely performed to identify disease-associated genes in AgP. GWAS enables the simultaneous analysis of several genes (10). Thus, this approach could open new frontiers in our understanding and treatment of AgP. This genomic analysis approach has been used to identify glycosyltransferase 6 domain containing 1 (*GLT6D1*) as an AgP-related gene in German and Dutch patients (11, 12). In addition, GWAS using German, Dutch, and Turkish AgP and CP patients identified SNPs in defensin α1 and α3 (*DEFA1A3*: rs2978951 and rs2738058) and sialic acid-binding Ig-like lectin 5 (*SIGLEC5*: rs4284742) as AgP-related genes (7). These GWAS identified AgP-associated variants using large-scale genetic studies of samples from patients of European descent. However, the limited ethnic background limits understanding of AgP's genetic architecture in non-European populations. The differences in allele frequencies across populations can be used to discover genetic markers that were not identified in European populations (13). To identify the AgP genetic risk factors in a Japanese population, we previously performed exome sequencing using DNA isolated from Japanese patients with AgP. The SNPs of ten genes were identified as AgP-related candidate genes, including rs536714306 G protein-coupled receptor 126 (*GPR126*). Functional analyses of *GPR126* revealed that PDL cells expressing SNP rs536714306 *GPR126* had decreased cAMP levels and expressed lower levels of calcification-related genes. This suggests that suppression of PDL cytodifferentiation by the SNP rs536714306 *GPR126* disrupts periodontal tissue homeostasis, thereby leading to the rapid destruction of the periodontal tissue (14).

In the previous study, we selected genes requiring “allele frequency less than 1% in the 1,000 Genomes Database”. However, this strict selection was disadvantageous for a small sample size because this criterion might reduce the statistical power (15). To eliminate this issue, in this study, we changed the filtering condition and re-analyzed the data from the whole exome sequencing to search for AgP-related genes more comprehensively.

PON-1 is a 43 kDa calcium-dependent glycoprotein with 355 amino acid residues (16, 17). It is synthesized in the liver, released into the circulation (18), and transported to several tissues (19). It binds to cell membranes and protects lipids against peroxidation (18). PON-1 prevents low-density lipoprotein oxidation and impairs the inflammatory response (20). It has been reported that the osteoblasts derived from *PON-1* transgenic mice had markedly increased the alkaline phosphatase (ALP) activity in the presence of parathyroid hormone, suggesting that PON-1 is involved in hard tissue-formation (21). Additionally, we have shown that the *PON-1* gene enhances the cytodifferentiation of periodontal ligament (PDL) cells into osteoblastic cells (22). However, it has not been elucidated whether the functions of SNP rs854560 in the *PON-1* gene locus was involved in the pathogenesis of AgP. Thus, in this study, we focused on the *PON-1* SNP rs854560 (mutant) among the identified AgP-related genes and further analyzed its functions in human periodontal ligament (HPDL) cells.

## 2 Materials and methods

### 2.1 Participants

We recruited 44 unrelated AgP patients who were diagnosed with AgP between the ages of 18–39 years old at Osaka University Dental Hospital (23). We examined the periodontal pocket depth and bleeding on probing at 6 sites around the teeth of the whole mouth. The periodontal inflamed surface area (PISA) was measured. Alveolar bone destruction from radiographic images was measured using the Schei ruler (24). The clinical attachment loss was defined if the alveolar bone loss was 30% or more by radiography. The clinical characteristics of the AgP patients were shown in Supplementary Table S1. All participants provided informed consent. This study was approved by the Osaka University Research Ethics Committee (approval no. 629). The patients were diagnosed as AgP based on the criteria from the American Academy of Periodontology's criteria.

### 2.2 Analysis of the whole exome sequencing for AgP-associated genes in a Japanese population

The materials and methods of the whole exome sequencing of the AgP patients were in Miyauchi et al. (23). Briefly, whole exome sequencing was performed (BGI Tech, Shenzhen, China) using the Illumina 2,000 or 4,000 platform (Illumina, San Diego, CA). To identify the genetic risk factors for AgP in the Japanese population, we obtained the data of the whole exome sequencing on the Japanese AgP patients from the DNA Data Bank of Japan at the National Institute of Genetics (JGAS 00000000024 and JGAS 00000000040). To comprehensively analyze AgP-related genes, we extended the threshold of the allele frequency from 1% to 5% and determined the selection criteria as follows: (i) variants with allele frequency ≤0.05 in the 1,000 Genome database, (ii) variants with read depths ≥10, (iii) variants that were present in four or more patients, and (iv) variants that caused protein structural and functional changes according to the results of *in silico* bioinformatic analysis using SIFT and Polyphen2. The AgP-related candidate genes were selected. Then, the variant allele frequency in AgP patients was compared with that in the control data from the Human Genetic Variation Database (HGVD), which includes 1,208 Japanese individuals (25). The controls from the database are without a clinical record associated with any major diseases. When the variant allele frequency in AgP was higher than that in the controls, we defined these variants as AgP-related suggestive genes.

### 2.3 Immunohistochemistry

The immunohistochemistry of PON-1 was performed in periodontal tissue in 8-week-old male C57BL6/J mice (Japan SLC, Hamamatsu, Japan). All experimental procedures confirmed to the guidelines of the Regulations for Animal Experiments and Related Activities at Osaka University and were reviewed by the Institutional Laboratory Animal Care and Use Committee of Osaka University (approval protocol No. R2-008). Briefly, mice were euthanized, perfused and fixed. After decalicification of the fixed maxillae, the tissues were embedded in SCEM (SECTION-LAB, Yokohama, Japan) and sagittally sectioned at 8 µm thickness with LEICA CM3050S (Leica Biosystems, IL) by the Kawamoto's Film Method (26). After fixing and blocking, the frozen sections were stained with PON1 Polyclonal antibody (Proteintech, Rosemont, IL) or Rabbit Ab IgG isotype control (Cell Signaling Technology, Danvers, MA), followed by Alexa Fluor546 goat anti-rabbit IgG (Life Technologies, Carlsbad, CA). The sections were stained with 4′, 6-diamidino-2-phenylindole (DAPI) and images were taken using Keyence BZ-X810 Fluorescence Microscope (KEYENCE, Osaka, Japan).

### 2.4 Cell culture

Human periodontal ligament (HPDL) cells were purchased from ScienCell Research Laboratories (Carlsbad, CA). The cells were cultured and incubated in α-modified Eagle's medium (Wako, Osaka, Japan) supplemented with 10% fetal calf serum. For the induction of HPDL cell mineralization, we used α-modified Eagle's medium supplemented with 10% fetal calf serum, 50 μg/ml of L-ascorbic acid (Wako), and 5 mmol/L of β-glycerophosphate (Sigma-Aldrich, St Louis, MO) as the calcification-inducing medium, as described previously (22).

### 2.5 Transfection

To compare the *PON-1* expression levels and its enzyme activity in SNP rs854560 (mutant; mut) *PON-1* and WT *PON-1*, a WT *PON-1-*expressing vector and a control empty vector (pCMV3; Sino Biological, Beijing, China) were transfected into HPDL cells using Lipofectamine 3,000 (Thermo Fisher Scientific, Waltham, MA). Then, SNP rs854560 was inserted using the PrimeSTAR Mutagenesis Basal Kit (Takara Bio, Shiga, Japan) as the manufacturer's instruction. Finally, the cells were cultured in the calcification-inducing medium for 4 days to induce cytodifferentiation. The HPDL cells transfected with an empty vector were used as a control (Ev).

### 2.6 PON-1 activity assay

PON-1 enzyme activity was measured as previously described with slight modifications (27) to assess whether PON-1 activity was altered in mut compared to WT. Briefly, the HPDL cells were serum-starved for 4 h and washed twice with PBS. The cells were then incubated with an assay mixture comprising 1 mmol/L 4-nitrophenyl acetate (Nacalai Tesque, Kyoto, Japan) and 1 mmol/L CaCl_2_ in 50 mmol/L Tris-HCl (pH 8.0) for 45 min at 25°C. The supernatants were collected, and absorbance was measured at 405 nm using a microplate reader (BioRad Laboratories, Hercules, CA).

### 2.7 Real-time polymerase chain reaction (PCR)

Two days after transfection, total RNA was extracted using RNA bee (Tel-Test Inc., Friendswood, TX) and treated with DNase I (Takara Bio). Next, the total RNA was reverse-transcribed to synthesize complementary DNA (cDNA) using a High-Capacity RNA-to-cDNA Kit (Thermo Fisher Scientific). Real-time PCR was performed using the Power PCR SYBR Master Mix (Applied Biosystems, Carlsbad, CA) with gene-specific PCR primers. The primer sequences are shown in Table 1.

**Table 1:** The primer sequences used in real-time PCR analysis.

Gene names		Sequences
Paraoxonase-1 *(PON-1)*	F	5′-TGAACCATCCAGATGCCAAGTC-3′
	R	5′-GCTCAGGTCCCACAGCAACA-3′
Alkaline phosphatase *(ALP)*	F	5′-GGACCATTCCCACGTCTTCAC-3′
	R	5′-CCTTGTAGCCAGGCCCATTG-3′
Collagen type I alpha 1 chain *(COL-1)*	F	5′-CCCGGGTTTCAGAGACAACTTC-3′
	R	5′-TCCACATGCTTTATTCCAGCAATC-3′
Runt-related transcription factor 2 *(RUNX2)*	F	5′-CACTGGCGCTGCAACAAGA -3′
	R	5′-CATTCCGGAGCTCAGCAGAATAA -3′
Osteocalcin *(OC)*	F	5′-CCCAGGCGCTACCTGTATCAA -3′
	R	5′-GGTCAGCCAACTCGTCACAGTC -3′
Hypoxanthine-guanine phosphoribosyltransferase *(HPRT)*	F	5′-GGCAGTATAATCCAAAGATGGTCAA-3′
	R	5′-GTCAAGGGCATATCCTACAACAAAC-3′

### 2.8 Alkaline phosphatase (ALP) activity assay

ALP activity was assessed as previously described (22). Briefly, HPDL cells were washed twice with PBS, sonicated in 0.01 M Tris-HCl (pH 7.4), and centrifuged at 12,000 rpm for 5 min at 4°C. Next, 1 M Tris-HCl (pH 9.0), 5 mm MgCl_2_ (Wako), and 50 mm p-nitrophenyl phosphate (Wako) were added to the supernatant. Absorbance was measured at 405 nm using a microplate reader (Bio-Rad Laboratories) and normalized through the total amount of DNA.

### 2.9 Bromodeoxyuridine (BrdU) assay

BrdU assay was performed using a BrdU assay kit (Merck, Darmstadt, Germany) as per the manufacture's instruction. The luminous intensity was measured using GLOMAX® (Promega, Madison, WI).

### 2.10 Statistical analysis

**Figure 1:**
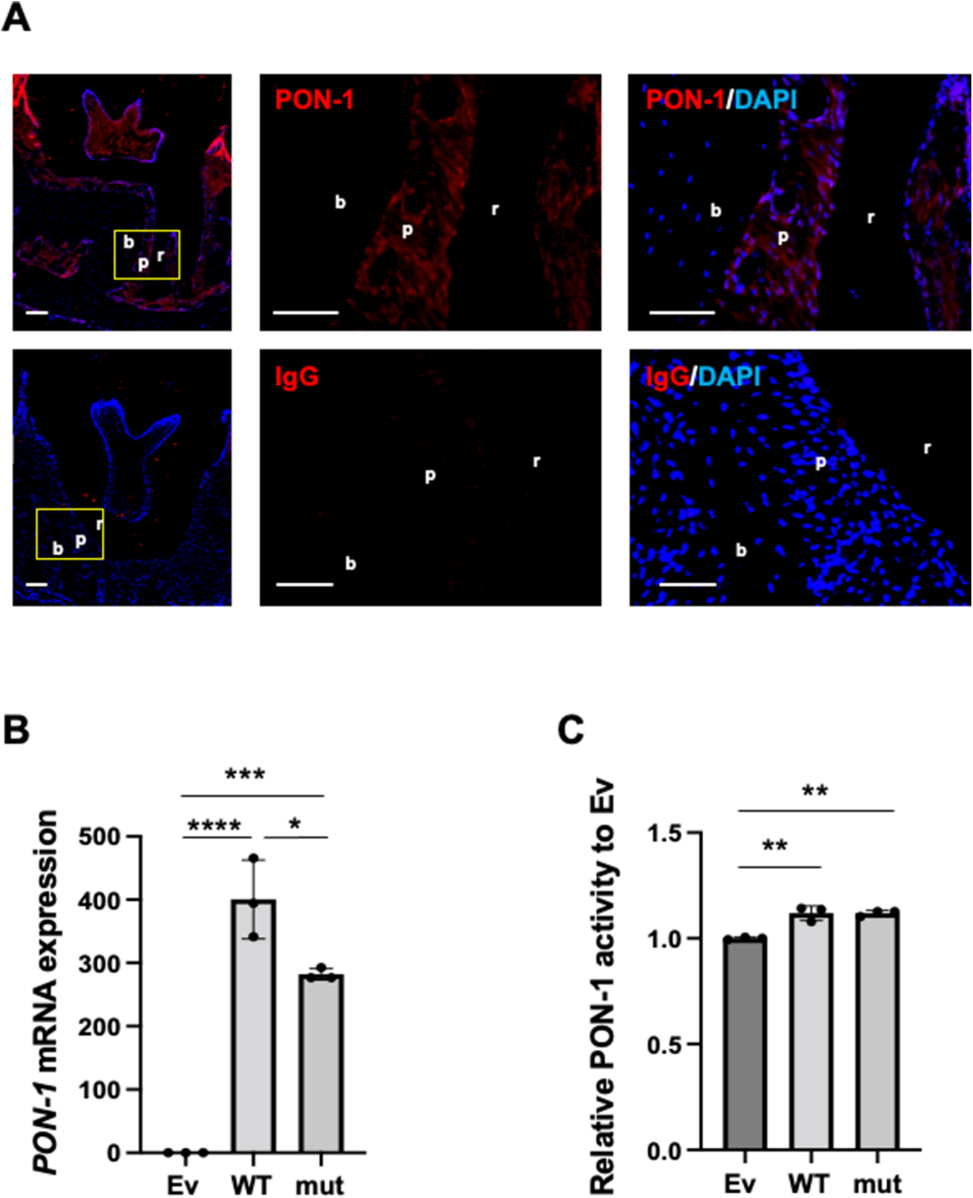
PON-1 expression and its activity in periodontal ligament. **(A)** PON-1 protein expression was analyzed in periodontal tissue around the 2nd molars in the wild-type mice by immunohistochemistry. *Upper panels*: PON-1; lower panels: isotype control. Red: PON-1 or isotype, blue: DAPI. The middle and right panels are the magnified images of the yellow area in the left panels. Scale bars: 100 µm in lower magnification (left); 50 µm in higher magnification (middle and right). r: root of a tooth; p: periodontal ligament; b: alveolar bone. Data are representative of three independent experiments. **(B)** Real-time PCR and **(C)** PON-1 activity assay in the Ev-, WT-, or mut-vector transfected into human periodontal ligament (HPDL) cells. Ev: empty vector, WT: wild-type *PON-1*, and mut: SNP rs854560 *PON-1*. PON-1 activity was normalized to that of Ev. Data represent the average and standard deviation (SD) of three independent experiments. *: *p* < 0.05, **: *p* < 0.01, ***: *p *< 0.001, ****: *p *< 0.0001.

**Figure 2:**
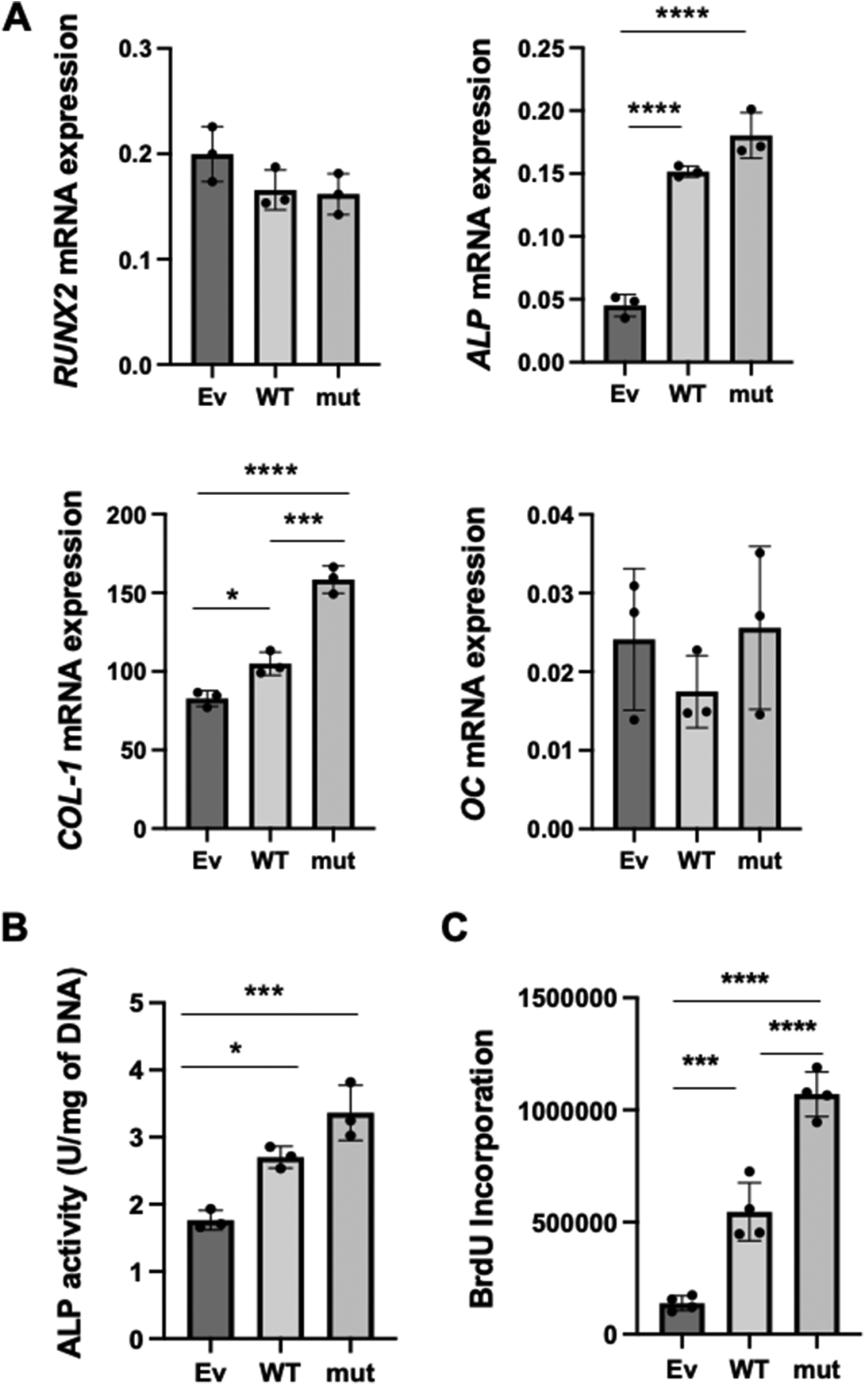
The effects of WT and SNP rs854560 *PON-1* on the cytodifferentiation and proliferation of HPDL cells. **(A)** The gene expression of *RUNX2*, *ALP*, *COL-1* and *OC* on day 4 of the cytodifferentiation of HPDL cells was assessed using real-time PCR. **(B)** ALP activity was measured on day 4 of the cytodifferentiation of HPDL cells. **(C)** The proliferation of HPDL cells was examined by BrdU assay. Ev: empty vector, WT: wild-type *PON-1* and mut: SNP rs854560 *PON-1*. Data represent the average and SD of three or four independent experiments. *: *p* < 0.05, ***: *p *< 0.001, ****: *p *< 0.0001.

A chi-square test was used for the data presented in Table 2 and Supplementary Table S2. One-way analysis of variance (ANOVA) was used for the data presented in Figures 1, 2. Statistical significance was set at *p *< 0.05.

**Table 2:** AgP-related suggestive genes in a Japanese population.

Gene name	dbSNP ID	*p*-value (AgP vs. HGVD)	AF of AgP (%)	AF of HGVD (%)	Chr	Position (bp)
*ENPP5*	rs16874326	1.6 × 10^−3^	10.2	3.6	6	46133282
*CCPG1*	rs34958422	5.0 × 10^−3^	10.2	4.1	15	55652719
*BCL2L14*	rs61739220	5.0 × 10^−3^	10.2	4.2	12	12247793
*SMPD3*	rs145616324	1.4 × 10^−2^	10.2	4.6	16	68405673
*PON-1*	rs854560	1.9 × 10^−2^	13.6	7.3	7	94946084
*CDK3*	rs34670267	2.1 × 10^−2^	5.7	2.0	17	73999328
*KAZALD1*	rs11547671	3.7 × 10^−2^	12.5	6.7	10	102822575

HGVD, human genetic variation database (the controls); AF, variant allele frequency (the percentage of SNP carriers in AgP or HGVD); Chr, chromosome.

## 3 Results

### 3.1 Analysis of the whole exome sequencing for AgP-associated suggestive genes in a Japanese population

Whole exome sequencing of 44 AgP patients found approximately 78,000 variants per individual. After we narrowed down the AgP-related candidate genes, 40 AgP-related candidate genes were identified. We further calculated the *p-*value of the variant allele frequency of each AgP-related candidate genes via comparison with the control data from the Human Genetic Variation Database (HGVD). The database includes 1,208 Japanese individuals without a clinical record associated with any major diseases (25). Our comprehensive filtering criteria enabled the identification of seven AgP-related suggestive genes whose *p*-value < 0.05 between the AgP group and the control groups (Table 2). In addition to these AgP-suggestive genes, we listed the rest of 33 AgP-related candidate genes (*p* ≥ 0.05) and the *p*-values of their variant allele frequencies between AgP and HGVD in Supplementary Table S2. Among the AgP-related suggestive genes, we selected the genes which have been reported to be associated with periodontitis and are expressed in the periodontal tissue, and found two genes, sphingomyelin phosphodiesterase 3 (*SMPD3*) and paraoxonase-1 (*PON-1*) (22, 23). Previously, we showed that the *SMPD3* SNP rs145616324 was involved in AgP by disturbing the homeostasis of periodontal tissue (23). Regarding *PON-1* SNP rs854560, a change in the nucleotide sequence at position 163th from thymine (T) to adenine (A) resulted in a change in its amino acid sequence at position 55th from leucine to methionine (28). The results of a previous study showed that carriers of the SNP rs854560 *PON-1* have a high rate of moderate periodontitis (22). PON-1 expression and its activity in periodontal ligament.

We analyzed PON-1 protein expression in periodontal tissue in mice by immunohistochemistry. Immunohistochemistry showed that PON-1 protein expression was detected in periodontal tissue as well as dental pulp and gingival epithelium (Figure 1A). Next, we accessed the *PON-1* mRNA expression and PON-1 activity in HPDL cells and compared them between wild-type (WT) *PON-1* and SNP rs854560 *PON-1*. We transfected WT or SNP rs854560 *PON-1*(mut) plasmids into HPDL cells. The empty vector (Ev)-transfected HPDL cells were used as a control. The real-time PCR analysis revealed that both WT and mut had higher *PON-1* mRNA expression compared to that in Ev (Figure 1B). We thus confirmed the success of transfection. Furthermore, mut showed lower *PON-1* mRNA expression than WT (Figure 1B). In the PON-1 activity assay, WT and mut showed considerably higher PON-1 activity than Ev, whereas no significant difference was observed between the WT and mut transfected HPDL cells (Figure 1C).

### 3.2 The effects of WT and SNP rs854560 *PON-1* on the cytodifferentiation and proliferation of HPDL cells

Previously, we demonstrated that *PON-1* was important for PDL cytodifferentiation (22). Therefore, we hypothesized that the SNP rs854560 *PON-1* regulated the cytodifferentiation of PDL cells to affect the pathogenesis of AgP. To verify our hypothesis, we examined the effects of SNP rs854560 *PON-1* on the cytodifferentiation of HPDL cells. We induced the cytodifferentiation of the transfected HPDL cells in the calcification-inducing media. Four days later, total RNA was collected and real-time PCR was performed to analyze mRNA expression of runt-related transcription factor 2 (*RUNX2*) (an early marker gene of cytodifferentiation), alkaline phosphatase (*ALP*), collagen type I alpha 1 chain (*COL-1*) (the marker genes of the middle stage of the cytodifferentiation) and osteocalcin (*OC*) (a late marker gene of the cytodifferentiation). We confirmed that the mRNA expression of *ALP* and *COL-1* was significantly upregulated in WT than Ev. Moreover, mut showed significantly higher mRNA expression of *COL-1* and a higher tendency of *ALP* mRNA expression compared to WT (Figure 2A). In contrast, mRNA expression of *RUNX2* and *OC* was equivalent between the WT and mut groups (Figure 2A). These results indicate that *PON-1* affected the middle stage of the cytodifferentiation of HPDL cells. Furthermore, we measured ALP activity in WT, mut, and Ev during cytodifferentiation of HPDL cells. WT and mut showed significantly higher ALP activity than Ev. Similar to the real-time PCR result, mut showed a higher tendency of ALP activity than WT (Figure 2B).

We further examined an effect of WT and SNP rs854560 *PON-1* on the proliferation of HPDL cells by BrdU assay. The result indicated that BrdU incorporation was significantly higher in WT and mut *PON-1* than Ev. In addition, mut showed significantly higher BrdU incorporation, compared to WT (Figure 2C).

## 4 Discussion

In this study, we identified seven AgP-related suggestive genes in a Japanese population using whole exome sequencing. Furthermore, we investigated the effects of SNP rs854560 *PON-1*, one of the AgP-related suggestive genes, on the functions of HPDL cells in comparison with WT *PON-1*. Through *in vitro-*experiments, we found that both HPDL cells expressing WT *PON-1* and mutant *PON-1* enhanced the cytodifferentiation and proliferation of HPDL cells. Additionally, HPDL cells expressing mutant *PON-1* showed higher mRNA expression levels of *ALP* and *COL-1*, ALP activity, and BrdU incorporation than those expressing WT *PON-1*. These results suggest that WT *PON-1* contributes to periodontal tissue homeostasis by promoting the appropriate proliferation and cytodifferentiation of HPDL cells. In contrast, SNP rs854560 *PON-1* may disrupt periodontal tissue homeostasis by inducing excessive calcification of PDL tissue due to hyper proliferation of HPDL cells, probably leading to the formation of fragile alveolar bone and increasing the risk of onset and progression of AgP.

As a result of the exome sequencing, we found several AgP-associated suggestive genes. Besides *PON-1* and *SMPD3*, there have been no reports directly showing the association between the identified genes and periodontitis. However, some of them are presumably related to AgP pathogenesis. For instance, cyclin-dependent kinase 3 (*CDK3*) belongs to the CDK family which regulates the phosphorylation of cyclins, resulting in cell cycle regulation (29).*CDK2/4/6* belonging to the same family as *CDK3* have been reported as disease-related variants for rheumatoid arthritis (30), which has similarities to periodontitis in terms of its manifestation of bone destruction via inflammation. Another AgP-candidate gene, ectonucleotide pyrophosphatase/phosphodiesterase family member 5 (*ENPP5*) belongs to the ENPP family and its expression and function(s) are mostly unknown. However, *ENPP1*, which is in the same subfamily as *ENPP5*, is known to be a causal gene in ttw (tip-tow walking) mice, which are used as animal models of ectopic calcification (31). Considering the functions of *ENPP1*, *ENPP5* might be involved in periodontal hard tissue-formation and in AgP onset and progression.

In this study, we used the database, HGVD, for the healthy controls and the results from the database showed the differences in the variant allele frequency between AgP and the controls. However, the control database contains no information on age, sex, and periodontal status because of ethical reasons. Moreover, the sample size of the controls is larger than that of the AgP group. These are the limitations of our study. To overcome the limitation, we performed the *in vitro* experiments and validated that the identified SNP was a causal variant and was involved in the pathogenesis of AgP. In the future, we need to statistically re-analyze the exome sequencing data using the equivalent sample size of the healthy controls who are not subjected to AgP.

In this study, we used the transfection method to examine the functions of SNP rs854560 *PON-1* because the endogenous expression of *PON-1* was detectable but not very high in the physiological condition, whereas it was gradually up-regulated during the cytodifferentiation (22). To mimic the status in the cytodifferentiation of PDL, the overexpressing the gene by transfection was a useful method. We used WT *PON-1* as a control for SNP rs854560 *PON-1*. By comparing the results in HPDL cells transfected with SNP rs854560 *PON-1* to those transfected with WT *PON-1*, we could understand the specific functions of SNP rs854560 *PON-1*.

PON-1 is a calcium-dependent esterase that degrades organophosphate compounds such as paraoxon (a potent cholinesterase inhibitor) and binds to high-density lipoproteins (HDLs) in the blood, inhibiting the oxidation of HDLs and low-density lipoproteins (32). Thus, PON-1 primarily possesses antioxidant properties. Hydrogen peroxide-induced oxidative stress reduces the expression of the *ALP* gene in osteoblasts (33). In contrast, the antioxidant docosahexaenoic acid increases the expression of *ALP* (34). An early study, in which *PON-1* overexpression was induced in hyperlipidemic mice *in vivo*, found an increase in the mRNA expression levels of *ALP* and osteoprotegerin and a decrease in the number of osteoclasts (21). These results suggest that *PON-1* promotes osteoblast differentiation and inhibits osteoclast formation.In a similar mechanism, we presume that SNP rs854560 *PON-1* in which elevated PON-1 activity in the PDL promotes the expression of ALP and enhances calcification in PDL. However, a mechanistic link between increased PDL calcification and alveolar bone loss observed in patients with AgP remains unclear. We speculate that the excessive calcification weaken the structural integrity of the PDL and surrounding alveolar bone, making it more susceptible to damage and loss in AgP. Further investigation is needed to validate this hypothesis by *in vivo* studies. Generating a transgenic mouse introducing SNP rs854560 *PON-1* will be instrumental in elucidating the exact role of PON-1 in PDL calcification and its subsequent impact on alveolar bone structural integrity.

As seen in Figure 2A, *PON-1* affected mRNA expression of *ALP* and *COL-1*, which are both marker genes of the middle stage of HPDL cytodifferentiation. In general, the lipid metabolism-related genes have a function to regulate the amount of the intracellular lipids (35). The intracellular lipids regulate adenosine triphosphate (ATP) production through oxidative phosphorylation in the mitochondria and fuel energy which is necessary for cytodifferentiation of osteoblasts (36, 37). At the mid-stage of cytodifferentiation of hard tissue-forming cells, many types of extracellular matrix genes are transcribed and translated (38). Furthermore, these proteins are packed in extracellular vesicles and intra/extracellularly transported (39). These processes require a considerable amount of energy. Thus, in addition to the antioxidant effects, *PON-1*, a lipid metabolism-related gene, may enhance the synthesis and production of the extracellular matrix proteins such as COL-1 at the mid-stage of HPDL cytodifferentiation by fueling the cells with increased ATP. Further studies are warranted to elucidate the detailed mechanisms of how *PON-1* affects the genes particularly expressed at the mid-stage of HPDL cytodifferentiation.

Previously, the presence of SNP rs854560 *PON-1* reduced the stability of the PON-1 protein (28). However, its mechanism was unclear. The amino acid glutamate at position 53th in the *PON-1* locus, and asparagine at the 54th position bind these calcium ions (40). In the SNP rs854560 *PON-1*, leucine is replaced by methionine at position 55th, near glutamate and asparagine (41). Thus, it was inferred that this SNP is also involved in the stability of PON-1 proteins.

The SNP rs145616324 in *SMPD3* among the AgP-related suggestive genes was previously reported to be associated with AgP (23). HDL cholesterol is lower in patients with congenital disorders of lipid metabolism, such as Niemann–Pick disease, which is caused by the deletion of sphingomyelin phosphodiesterase 1 (*SMPD1*), another member of the *SMPD* family (42). Moreover, SNPs rs120074118 and rs120074127 in *SMPD1* are closely associated with lower HDL cholesterol levels (43). As PON-1 binds to HDL cholesterol, presumably *PON-1* and the *SMPD* family could be mutually involved in the onset and progression of AgP. However, polymorphism analysis using data from 44 Japanese patients with AgP identified no patient with both SNP rs854560 *PON-1* and SNP rs145616324 *SMPD3*, although 12 AgP patients possessed the SNP rs854560 *PON-1* (163 subjects in HGVD possessed the SNP rs854560 *PON-1*) and 7 AgP patients possessed the SNP rs145616324 *SMPD3* (100 subjects in HGVD possessed the SNP rs145616324 *SMPD3*) (22, 23). This could be because only a small number of samples contained either SNP. Nonetheless, further studies on disease-associated genes are required to provide a better understanding of AgP because AgP traits are supposedly developed by a group of these AgP-associated genes.

The main challenge of this study is to recruit sufficient Japanese AgP patients because the prevalence of AgP in the Japanese population is only 0.03% (44). Generally, to identify disease-related genes by GWAS requires approximately 10,000 subjects (45).In this study, we could not identify the variants meeting genome-wide significance (*p* < 5.0 × 10^−8^) mainly because the simple size of the AgP patients was too small (*n* = 44) compared to the standard sample size of GWAS. To overcome this issue, we are increasing the sample size by collaborating with dental clinics and institutes in Japan to clarify the precise proportion of patients who possess a combination of AgP-causal variants.

In conclusion, we identified seven AgP-related suggestive genes, including SNP rs854560 *PON-1* from the exome sequencing data with extended filtration conditions. WT *PON-1* maintained the homeostasis in the periodontal tissue by inducing the cytodifferentiation of PDL cells. In contrast, SNP rs854560 *PON-1* induced excess proliferation of HPDL cells and ALP activity and mRNA expression of calcification-related genes during the cytodifferentiation of HPDL cells, probably resulting in the induction of hypercalcification of the PDL. These results suggest that the SNP rs854560 *PON-1* disturbs the homeostasis of periodontal tissue and accelerates AgP progression. In the future, we expect that SNP rs854560 *PON-1* will be used as a risk marker to predict AgP onset and progression and provide preventive interventions for patients with pre-AgP.

## Data Availability

Publicly available datasets were analyzed in this study. The exome sequencing datasets analyzed for this study can be found in the DNA Data Bank of Japan at the National Institute of Genetics (JGAS 00000000024 and JGAS 00000000040); https://humandbs.biosciencedbc.jp/en/hum0027-v1.

## References

[1] de Jong T Bakker AD Everts V Smit TH. The intricate anatomy of the periodontal ligament and its development: lessons for periodontal regeneration. J Periodontal Res. (2017) 52(6):965–74. 10.1111/jre.12477 28635007

[2] Zhai Q Dong Z Wang W Li B Jin Y. Dental stem cell and dental tissue regeneration. Front Med. (2019) 13(2):152–9. 10.1007/s11684-018-0628-x 29971640

[3] Albandar JM. Aggressive and acute periodontal diseases. Periodontol 2000. (2014) 65(1):7–12. 10.1111/prd.12013 24738583

[4] Brito LF Taboza ZA Silveira VR Furlaneto FA Rosing CK Rego RO. Aggressive periodontitis presents a higher degree of bilateral symmetry in comparison with chronic periodontitis. J Oral Sci. (2018) 60(1):97–104. 10.2334/josnusd.16-0669 29576581

[5] Shimizu S Momozawa Y Takahashi A Nagasawa T Ashikawa K Terada Y A genome-wide association study of periodontitis in a Japanese population. J Dent Res. (2015) 94(4):555–61. 10.1177/0022034515570315 25672891

[6] Sanders AE Sofer T Wong Q Kerr KF Agler C Shaffer JR Chronic periodontitis genome-wide association study in the Hispanic community health study/study of Latinos. J Dent Res. (2017) 96(1):64–72. 10.1177/0022034516664509 27601451 PMC5347427

[7] Munz M Willenborg C Richter GM Jockel-Schneider Y Graetz C Staufenbiel I A genome-wide association study identifies nucleotide variants at SIGLEC5 and DEFA1A3 as risk loci for periodontitis. Hum Mol Genet. (2018) 27(5):941–2. 10.1093/hmg/ddy015 29346566

[8] Loos BG Van Dyke TE. The role of inflammation and genetics in periodontal disease. Periodontol 2000. (2020) 83(1):26–39. 10.1111/prd.12297 32385877 PMC7319430

[9] Golan D Lander ES Rosset S. Measuring missing heritability: inferring the contribution of common variants. Proc Natl Acad Sci U S A. (2014) 111(49):E5272–81. 10.1073/pnas.1419064111 25422463 PMC4267399

[10] Weissenkampen JD Jiang Y Eckert S Jiang B Li B Liu DJ. Methods for the analysis and interpretation for rare variants associated with complex traits. Curr Protoc Hum Genet. (2019) 101(1):e83. 10.1002/cphg.83 30849219 PMC6455968

[11] Hashim NT Linden GJ Ibrahim ME Gismalla BG Lundy FT Hughes FJ Replication of the association of GLT6D1 with aggressive periodontitis in a Sudanese population. J Clin Periodontol. (2015) 42(4):319–24. 10.1111/jcpe.12375 25682733

[12] Schaefer AS Richter GM Nothnagel M Manke T Dommisch H Jacobs G A genome-wide association study identifies GLT6D1 as a susceptibility locus for periodontitis. Hum Mol Genet. (2010) 19(3):553–62. 10.1093/hmg/ddp508 19897590

[13] Ishigaki K Akiyama M Kanai M Takahashi A Kawakami E Sugishita H Large-scale genome-wide association study in a Japanese population identifies novel susceptibility loci across different diseases. Nat Genet. (2020) 52(7):669–79. 10.1038/s41588-020-0640-3 32514122 PMC7968075

[14] Kitagaki J Miyauchi S Asano Y Imai A Kawai S Michikami I A putative association of a single nucleotide polymorphism in GPR126 with aggressive periodontitis in a Japanese population. PLoS One. (2016) 11(8):e0160765. 10.1371/journal.pone.0160765 27509131 PMC4979892

[15] Guo MH Dauber A Lippincott MF Chan YM Salem RM Hirschhorn JN. Determinants of power in gene-based burden testing for monogenic disorders. Am J Hum Genet. (2016) 99(3):527–39. 10.1016/j.ajhg.2016.06.031 27545677 PMC5011058

[16] Draganov DI Stetson PL Watson CE Billecke SS La Du BN. Rabbit serum paraoxonase 3 (PON3) is a high density lipoprotein-associated lactonase and protects low density lipoprotein against oxidation. J Biol Chem. (2000) 275(43):33435–42. 10.1074/jbc.M004543200 10931838

[17] Lu H Zhu J Zang Y Ze Y Qin J. Cloning, purification, and refolding of human paraoxonase-3 expressed in Escherichia coli and its characterization. Protein Expr Purif. (2006) 46(1):92–9. 10.1016/j.pep.2005.07.021 16139510

[18] Deakin S Leviev I Gomaraschi M Calabresi L Franceschini G James RW. Enzymatically active paraoxonase-1 is located at the external membrane of producing cells and released by a high affinity, saturable, desorption mechanism. J Biol Chem. (2002) 277(6):4301–8. 10.1074/jbc.M107440200 11726658

[19] Marsillach J Mackness B Mackness M Riu F Beltrán R Joven J Immunohistochemical analysis of paraoxonases-1, 2, and 3 expression in normal mouse tissues. Free Radic Biol Med. (2008) 45(2):146–57. 10.1016/j.freeradbiomed.2008.03.023 18440321

[20] Navab M Imes SS Hama SY Hough GP Ross LA Bork RW Monocyte transmigration induced by modification of low density lipoprotein in cocultures of human aortic wall cells is due to induction of monocyte chemotactic protein 1 synthesis and is abolished by high density lipoprotein. J Clin Invest. (1991) 88(6):2039–46. 10.1172/JCI115532 1752961 PMC295797

[21] Lu J Cheng H Atti E Shih DM Demer LL Tintut Y. Role of paraoxonase-1 in bone anabolic effects of parathyroid hormone in hyperlipidemic mice. Biochem Biophys Res Commun. (2013) 431(1):19–24. 10.1016/j.bbrc.2012.12.114 23291186 PMC3563775

[22] Masumoto R Kitagaki J Matsumoto M Miyauchi S Fujihara C Yamashita M Effects of paraoxonase 1 on the cytodifferentiation and mineralization of periodontal ligament cells. J Periodontal Res. (2018) 53(2):200–9. 10.1111/jre.12507 29063603

[23] Miyauchi S Kitagaki J Masumoto R Imai A Kobayashi K Nakaya A Sphingomyelin phosphodiesterase 3 enhances cytodifferentiation of periodontal ligament cells. J Dent Res. (2017) 96(3):339–46. 10.1177/0022034516677938 28221099

[24] Schei O Waerhaung J Lovdal A Arno A. Alveolar bone loss as related to oral hygiene and age. J Periodont. (1959) 30:7–15. 10.1902/jop.1959.30.1.7

[25] Higasa K Miyake N Yoshimura J Okamura K Niihori T Saitsu H Human genetic variation database, a reference database of genetic variations in the Japanese population. J Hum Genet. (2016) 61(6):547–53. 10.1038/jhg.2016.12 26911352 PMC4931044

[26] Kawamoto T Kawamoto K. Preparation of thin frozen sections from nonfixed and undecalcified hard tissues using Kawamoto's film method. Methods Mol Biol. (2021) 2230:259–81. 10.1007/978-1-0716-1028-2_15 33197019

[27] Ceron JJ Tecles F Tvarijonaviciute A. Serum paraoxonase 1 (PON1) measurement: an update. BMC Vet Res. (2014) 10:74. 10.1186/1746-6148-10-74 24666514 PMC3985595

[28] Grzegorzewska AE Mostowska A Warchoł W Jagodziński PP. Paraoxonase 1 gene (PON1) variants concerning hepatitis C virus (HCV) spontaneous clearance in hemodialysis individuals: a case-control study. BMC Infect Dis. (2021) 21(1):875. 10.1186/s12879-021-06597-4 34445971 PMC8394142

[29] Łukasik P Baranowska-Bosiacka I Kulczycka K Gutowska I. Inhibitors of cyclin-dependent kinases: types and their mechanism of action. Int J Mol Sci. (2021) 22(6):2806. 10.3390/ijms22062806 33802080 PMC8001317

[30] Okada Y Wu D Trynka G Raj T Terao C Ikari K Genetics of rheumatoid arthritis contributes to biology and drug discovery. Nature. (2014) 506(7488):376–81. 10.1038/nature12873 24390342 PMC3944098

[31] Watanabe R Fujita N Sato Y Kobayashi T Morita M Oike T Enpp1 is an anti-aging factor that regulates klotho under phosphate overload conditions. Sci Rep. (2017) 7(1):7786. 10.1038/s41598-017-07341-2 28798354 PMC5552841

[32] Lioudaki S Verikokos C Kouraklis G Ioannou C Chatziioannou E Perrea D Paraoxonase-1: characteristics and role in atherosclerosis and carotid artery disease. Curr Vasc Pharmacol. (2019) 17(2):141–6. 10.2174/1570161115666171129212359 29189170

[33] Jung WW. Protective effect of apigenin against oxidative stress-induced damage in osteoblastic cells. Int J Mol Med. (2014) 33(5):1327–34. 10.3892/ijmm.2014.1666 24573323

[34] Cifuentes-Mendiola SE Moreno-Fierros L Gonzalez-Alva P Garcia-Hernandez AL. Docosahexaenoic acid improves altered mineralization proteins, the decreased quality of hydroxyapatite crystals and suppresses oxidative stress induced by high glucose. Exp Ther Med. (2022) 23(3):235. 10.3892/etm.2022.11160 35222712 PMC8815046

[35] Li F Zhang H. Lysosomal acid lipase in lipid metabolism and beyond. Arterioscler Thromb Vasc Biol. (2019) 39(5):850–6. 10.1161/ATVBAHA.119.312136 30866656 PMC6482091

[36] Fujihara C Yamada S Ozaki N Takeshita N Kawaki H Takano-Yamamoto T Role of mechanical stress-induced glutamate signaling-associated molecules in cytodifferentiation of periodontal ligament cells. J Biol Chem. (2010) 285(36):28286–97. 10.1074/jbc.M109.097303 20576613 PMC2934693

[37] Nantakeeratipat T Fujihara C Nogimori T Matsumoto M Yamamoto T Murakami S. Lysosomal acid lipase regulates bioenergetic process during the cytodifferentiation of human periodontal ligament cells. Biochem Biophys Res Commun. (2023) 662:84–92. 10.1016/j.bbrc.2023.04.041 37099814

[38] Nagayasu-Tanaka T Anzai J Takaki S Shiraishi N Terashima A Asano T Action mechanism of fibroblast growth factor-2 (FGF-2) in the promotion of periodontal regeneration in beagle dogs. PLoS One. (2015) 10(6):e0131870. 10.1371/journal.pone.0131870 26120833 PMC4488280

[39] Wei Y Tang C Zhang J Li Z Zhang X Miron RJ Extracellular vesicles derived from the mid-to-late stage of osteoblast differentiation markedly enhance osteogenesis in vitro and in vivo. Biochem Biophys Res Commun. (2019) 514(1):252–8. 10.1016/j.bbrc.2019.04.029 31029430

[40] Harel M Aharoni A Gaidukov L Brumshtein B Khersonsky O Meged R Structure and evolution of the serum paraoxonase family of detoxifying and anti-atherosclerotic enzymes. Nat Struct Mol Biol. (2004) 11(5):412–9. 10.1038/nsmb767 15098021

[41] Abudayyak M Boran T Tukel R Oztas E Özhan G. The role of PON1 variants in disease susceptibility in a turkish population. Glob Med Genet. (2020) 7(2):41–6. 10.1055/s-0040-1715568 32939514 PMC7490120

[42] Acuña M Martínez P Moraga C He X Moraga M Hunter B Epidemiological, clinical and biochemical characterization of the p.(Ala359Asp) SMPD1 variant causing niemann-pick disease type B. Eur J Hum Genet. (2016) 24(2):208–13. 10.1038/ejhg.2015.89 25920558 PMC4717211

[43] Lee CY Krimbou L Vincent J Bernard C Larramee P Genest J Jr. Compound heterozygosity at the sphingomyelin phosphodiesterase-1 (SMPD1) gene is associated with low HDL cholesterol. Hum Genet. (2003) 112(5-6):552–62. 10.1007/s00439-002-0893-1 12607113

[44] Takahashi M Chen Z Watanabe K Kobayashi H Nakajima T Kimura A Toll-like receptor 2 gene polymorphisms associated with aggressive periodontitis in Japanese. Open Dent J. (2011) 5:190–4. 10.2174/1874210601105010190 22235236 PMC3253990

[45] Shaffer JR Feingold E Marazita ML. Genome-wide association studies: prospects and challenges for oral health. J Dent Res. (2012) 91(7):637–41. 10.1177/0022034512446968 22562461 PMC3383848

