# Supplementary material for: Single nucleotide polymorphism rs854560 in paraoxonase-1 regulates the cytodifferentiation of human periodontal ligament cells
Source: Front Dent Med. 2024 Sep 20;5:1449482. doi: 10.3389/fdmed.2024.1449482 (PMC11797752; doi:10.3389/fdmed.2024.1449482)
Supplement: Supplementary file 1 [file Table1.docx]

Supplementary Material

Single nucleotide polymorphism rs854560 in paraoxonase-1 regulates the cytodifferentiation of human periodontal ligament cells.

Risa Masumoto^1†^, Chiharu Fujihara^1†*^, Masahiro Matsumoto^1^, Jirouta Kitagaki^1^, Shinya Murakami^1^

*** Correspondence:** Chiharu Fujihara: [fujihara.chiharu.dent@osaka-u.ac.jp](mailto:fujihara.chiharu.dent@osaka-u.ac.jp)

# Supplementary Tables

**Supplementary table 1. The clinical characteristics of the AgP participants**

Characteristics AgP patients (n=44)

Age (years) 32.55 $\pm$ 6.77

Sex Male: 15, Female: 29

Number of present teeth 27.91 $\pm$ 1.85

Probing pocket depth (mm) 4.18 $\pm$ 1.23

PISA (mm^2^) 1302.96 $\pm$ 1065.29

Alveolar bone resorption (%) 37.47 $\pm$ 15.43

PISA: periodontal inflamed surface area

Data represent the mean $\pm$standard deviation
