# Supplementary material for: Single nucleotide polymorphism rs854560 in paraoxonase-1 regulates the cytodifferentiation of human periodontal ligament cells
Source: Front Dent Med. 2024 Sep 20;5:1449482. doi: 10.3389/fdmed.2024.1449482 (PMC11797752; doi:10.3389/fdmed.2024.1449482)
Supplement: Supplementary file 2 [file Table2.docx]

Supplementary Material

Single nucleotide polymorphism rs854560 in paraoxonase-1 regulates the cytodifferentiation of human periodontal ligament cells.

Risa Masumoto^1†^, Chiharu Fujihara^1†*^, Masahiro Matsumoto^1^, Jirouta Kitagaki^1^, Shinya Murakami^1^

*** Correspondence:** Chiharu Fujihara: [fujihara.chiharu.dent@osaka-u.ac.jp](mailto:fujihara.chiharu.dent@osaka-u.ac.jp)

**Supplementary table 2. The additional AgP-related candidate genes**

| **Gene name** | **dbSNP ID** | ***p*-value** | **AF of AgP (%)** | **AF of HGVD (%)** | **Chr** | **Position (bp)** |
| --- | --- | --- | --- | --- | --- | --- |
| ***MIIP*** | **rs11553925** | **0.148** | **6.8** | **3.8** | **1** | **12082334** |
| ***MIIP*** | **rs11588712** | **0.082** | **6.8** | **3.4** | **1** | **12082461** |
| ***EPHA8*** | **rs76452513** | **0.389** | **12.5** | **9.7** | **1** | **22915858** |
| ***DENND4B*** | **rs35902206** | **0.0773** | **9.1** | **17.7** | **1** | **153907305** |
| ***DENND4B*** | **rs2275483** | **0.126** | **11.4** | **17.9** | **1** | **153907306** |
| ***WWTR1*** | **rs112399999** | **0.824** | **19.3** | **20.3** | **3** | **149238595** |
| ***DCHS2*** | **rs28561984** | **0.0998** | **9.1** | **9.3** | **4** | **155219318** |
| ***EGFLAM*** | **rs192113610** | **0.188** | **5.7** | **3.3** | **5** | **38370556** |
| ***C6*** | **rs77732988** | **0.770** | **8.0** | **7.2** | **5** | **41142932** |

| ***C6*** | **rs76528010** | **0.776** | **8.0** | **7.2** | **5** | **41159192** |  |
| --- | --- | --- | --- | --- | --- | --- | --- |
| ***C6*** | **rs80108105** | **0.786** | **8.0** | **7.2** | **5** | **41159339** |  |
| ***CDSN*** | **rs117951780** | **0.387** | **9.1** | **12.0** | **6** | **31084034** |  |
| ***PSORS1C1*** | **rs138474986** | **0.110** | **10.2** | **15.0** | **6** | **31106500** |  |
| ***CD164*** | **rs147143577** | **0.545** | **9.1** | **7.4** | **6** | **109689521** |  |
| ***TRIM4*** | **rs76665876** | **0.488** | **11.4** | **9.2** | **7** | **99489857** |  |
| ***CDHR3*** | **rs73195662** | **0.368** | **8.0** | **5.7** | **7** | **105658460** |  |
| ***SLCO5A1*** | **rs117676215** | **0.220** | **11.4** | **7.7** | **8** | **70585325** |  |
| ***NIPAL2*** | **rs77774174** | **0.143** | **20.5** | **14.8** | **8** | **99234772** |  |
| ***TJP2*** | **rs41305539** | **0.0768** | **13.6** | **8.3** | **9** | **71835842** |  |
| ***CYP2C18*** | **rs41271550** | **0.306** | **11.4** | **8.3** | **10** | **96447562** |  |
| ***CYP2C19*** | **rs4986893** | **0.306** | **11.4** | **8.3** | **10** | **96540410** |  |
| ***OR6C75*** | **rs75456529** | **0.457** | **5.7** | **7.8** | **12** | **55759192** |  |
| ***ACSS3*** | **rs61745251** | **0.516** | **13.6** | **11.4** | **12** | **81528625** |  |
| ***COQ7*** | **rs77337400** | **0.271** | **8.0** | **5.3** | **16** | **19083312** |  |
| ***CES1*** | **rs3826192** | **0.637** | **15.9** | **14.1** | **16** | **55862824** |  |
| ***MT1E*** | **rs141266321** | **0.832** | **9.1** | **8.5** | **16** | **56659783** |  |
| ***GLOD4*** | **rs75027378** | **0.637** | **9.1** | **7.7** | **17** | **663468** |  |
| ***KCNJ12*** | **rs1714865** | **0.994** | **8.0** | **8.0** | **17** | **21318821** |  |
| ***GEMIN4*** | **rs61753060** | **0.793** | **6.8** | **7.4** | **17** | **650510** |  |
| ***RNTML1*** | **rs75658007** | **0.349** | **9.1** | **13.1** | **17** | **695061** |  |
| ***RNTML1*** | **rs80220493** | **0.369** | **9.1** | **6.6** | **17** | **695062** |  |
| ***PLIN5*** | **rs1062223** | **0.983** | **4.5** | **4.6** | **19** | **4524016** |  |
| ***CSNK1E*** | **rs77945315** | **0.502** | **10.2** | **8.2** | **22** | **38689321** |  |

HGVD: Human Genetic Variation Database (the controls)

AF: variant allele frequency (the percentage of SNP carriers in AgP or HGVD)

Chr: chromosome

The variants detected in HGVD were listed in the table.

*p*-value (Chi-square test) : AgP vs HGVD
